# Assessment of the effectiveness of the peptide inhibitor homologous to the transforming growth factor β cytokine blocking the TGFβRI/TGFβRII receptor complex—pilot study

**DOI:** 10.1002/clt2.12320

**Published:** 2023-12-31

**Authors:** Marynowski Mateusz, Karbownik Michał Seweryn, Szemraj Janusz, Kuna Piotr, Michał Gabriel Panek

**Affiliations:** ^1^ Department of Internal Medicine, Asthma and Allergy Medical University of Lodz Lodz Lodzkie Poland; ^2^ Department of Pharmacology and Toxicology Medical University of Lodz Lodz Lodzkie Poland; ^3^ Department of Medical Biochemistry Medical University of Lodz Lodz Poland

**Keywords:** peptide inhibitors, SEAP reporter gene, the cytokine TGF‐β, the MFB‐F11 line, the TGFβRI/TGFβRII receptor

## Abstract

**Background:**

A key player in the fibrotic process is the transforming growth factor β (TGF‐β) which enhances extracellular matrix production by increasing the transcription of matrix proteins. The cytokine TGF‐β first binds to the TGFβRII receptor (dimer), resulting in the recruitment of the TGFβRI receptor (dimer). The complex thus formed leads to the phosphorylation of the kinase domain of TGFβRI, which in turn results in activation of the Smad pathway. This is therefore a targeted pathway for research into the application of peptide inhibitors in blocking the TGF‐β‐Smad signaling pathway. The aim of this study was to design a peptide inhibitor (homologous to the cytokine TGF‐β) which, after binding to the TGFβRI/TGFβRII receptor, would block the cytokine binding and thus prevent the formation of an activating complex.

**Methods:**

Preliminary work on the design and synthesis of inhibitors for TGFβRI/TGFβRII has allowed us to identify and describe five key regions of the TGF‐β—TGFβRI/TGFβRII interface. The following five peptide inhibitors were synthesized for Region 1: 1.1 ALDAAYCFR, 1.2 LDAAYCFRN, 1.3 DAAYCFRNV, 1.4 AAYCFRNVQ, 1.5 AYCFRNVQD. The expression of the SEAP reporter gene, Smad2, Smad3, Smad4, and JNK1 gene was measured using quantitative real‐time polymerase chain reaction.

**Results:**

For Region 1 peptide inhibitors tested for TGFβRI/TGFβRII, reduced SEAP (reporter gene) expression was observed in cells of the MFB‐F11 line, which suggests inhibited the formation of cytokine‐receptor complexes.

**Conclusions:**

For IP1_2, 1_3 and 1_5 Region 1 peptides tested for TGFβRI/TGFβRII, reduced cytokine‐receptor signal by adding newly designed inhibitors. The study revealed an impact of these peptide inhibitors on the reduction of mRNA expression of Smad2, Smad3, Smad4 and JNK1 genes.

## 1 INTRODUCTION

The progressive fibrosis in the kidneys, liver, heart, lung, bone marrow, and skin is a major cause of morbidity and mortality. A key player in this process is the transforming growth factor β (TGF‐β), which enhances extracellular matrix production by both increasing the transcription of matrix proteins, for example, fibronectin and collagen, and inhibiting enzymes responsible for matrix degradation. The initiation and development of inflammation and microdamage to respiratory epithelium is strongly regulated by the TGF‐β‐Smad (the *Drosophila* gene “mothers against decapentaplegic” (Mad) and the *C. elegans* gene Sma) protein pathway.^1^, ^2^

TGF‐β is a pleiotropic cytokine that regulates cell proliferation, contributes to epithelial‐to‐mesenchymal transition (EMT), suppresses the function of immune cells compromising the immune response, contributes to conversion of fibroblasts to myofibroblasts and causes overproduction of extracellular matrix (ECM) in tissues undergoing fibrosis. TGF‐β upregulates the expression and synthesis of many matrix proteins, primarily through the recruitment of myofibroblasts. Proteins upregulated by TGF‐β include collagens I‐V, basement membrane proteins (laminin, entactin, perlecan) and ECM proteins (fibronectin, osteopontin, thrombospontin, tenascin, osteonectin/SPARC, elastin, biglycan, decorin, and hyaluronan). Additionally, in the early stages of fibrosis, TGF‐β stimulates myofibroblasts and other stromal cells to enhance the synthesis of collagen crosslinking enzymes, particularly lysyl oxidase, which increases the rigidity of the collagen network. Moreover, TGF‐β downregulates the synthesis of matrix‐depleting proteins, such as matrix metalloproteinases (MMP‐1, ‐8, ‐13). As a result, the increase in matrix protein synthesis and decrease in matrix proteinase activity, owing to the TGF‐β activity, contribute to remodeling of the ECM and can result in a fibrotic response.^3^, ^4^, ^5^

Receptors for TGF‐β are present in all types of human cells, which means that the entire TGF‐β superfamily plays a significant role in the regulation of immune mechanisms. There are three recognized types of membrane receptors for this cytokine: TGFβRI, TGFβRII and TGFβRIII. Seven subgroups (Alk1‐Alk7) can be distinguished within the TGFβRI receptor (the so called activin receptor‐like kinase 5, Alk5). The TGF‐β family first of all binds to TGFβRII, which leads to recruitment of TGFβRI and subsequently to formation of TGFβRI/TGFβRII dimer. The complex thus created is responsible for the phosphorylation of the TGFβRI kinase domain, which in turn results in the activation of the Smad pathway. Activation and stabilization of TGFβRI/TGFβRII are regulated through posttranslational modifications, such as phosphorylation, ubiquitylation, sumoylation, and neddylation. The dimer of receptors I and II is also controlled by interactions with other proteins at the cell surface and in the cytoplasm. Activation of TGFβRI/TGFβRII through TGF‐β stimulates the signaling pathway via the formation of Smad complexes that are translocated to the nucleus where they act as transcription factors. This process may also take place via non‐Smad pathways, in which the Erk1/2, Jun N‐terminal kinase (JNK) and p38 MAP kinase pathways, Src tyrosine kinase, phosphatidylinositol 3′‐kinase, and Rho GTPases are also involved.^3^, ^4^, ^5^, ^6^, ^7^, ^8^, ^9^

Hyperactivity of TGF‐β‐Smad pathway by specific TGFβRI/TGFβRII receptors (two highly conserved single transmembrane receptors with intracellular serine/threonine kinase domains) leads to the development of chronic pathway inflammation and miofibroblast activation. Therefore, the inhibition of TGFβRI (also known as activin receptor‐like kinase 5, Alk5) seems to be a good strategy for the treatment of many abovementioned disorders manifesting with fibrosis.^9^, ^10^, ^11^

To sum up, TGF‐β is a pluripotent cytokine involved in a range of cellular functions including cell growth, proliferation, differentiation and death. The TGF‐β‐Smad signaling pathway is also muddled in the pathophysiology of many diseases including organ fibrosis, such as asthma or COVID‐19 (Coronavirus disease), but also plays a significant role in the development of several cancers.

Research has been conducted on the use of monoclonal antibodies and small‐molecule and peptide inhibitors in blocking the TGF‐β‐Smad signaling pathway. Various studies confirm that small‐molecule inhibitors such as SD‐208, and SB‐525334 have such potential. SD‐208 is a small‐molecule, selective TGFβRI inhibitor, and it prevents Smad‐2 phosphorylation by binding to the kinase of this receptor and maintaining it in an inactive configuration. It has been shown to inhibit TGF‐β1 and TGF‐β2 cytokine‐stimulated cell growth and migration, and to reduce the effect of these cytokines on decreasing susceptibility of malignant tumor cells to the immune system, both in vivo and in vitro. SD‐208 also inhibits the effects of TGF‐β1 on physiologically occurring cells, such as vascular endothelial muscle cells, reducing their proliferation and migration, and smooth muscle and cup cells in the airways, reducing their TGF‐β1‐induced proliferation during exposure to allergens.^12^, ^13^, ^14^, ^15^ SB‐525334 is an inhibitor of TGFβRI. Its selectivity toward TGFβRI and its inhibitory effect on phosphorylation and translocation of Smad signaling proteins to the cell nucleus and TGF‐β1‐stimulated gene transcription have been confirmed. These pharmacodynamic properties enabled SB‐525334 to show an inhibitory effect on carcinogenesis in a cellular model of ovarian cancer, reducing the division potential of tumor cells and their ability to migrate and infiltrate.^16^, ^17^

Many different studies reveal that substances (including those with pharmacological/therapeutic potential) such as small‐molecule or peptide inhibitors of TGFβRI/II may have therapeutic potential. However, it should be pointed out that each class of these molecules is characterized with certain limitations regarding specificity and toxicity as well as general limitations manifesting with deleterious off‐target effects, individualized drug efficacy and contextual dependency on cancer cell types or non‐cancer cell types and clinical stages.^18^, ^19^, ^20^, ^21^

## 2 AIM

The aim of this study was to design a PI (homologous to the cytokine TGF‐β) which, after binding to the TGFβRI/TGFβRII receptor, would block the cytokine binding and thus prevent the formation of an activating complex.

## 3 MATERIAL AND METHODS

1. Determination of the minimum TGF‐β—TGFβRI and TGF‐β—sTGFβRII interface

Based on manual inspection of the crystallographic structure of the TGFβRI, TGFβRII, TGF‐β1 complex (https://www.wwpdb.org, The Protein Data Bank, PDB: 3KFD: Ternary complex of TGF‐β1 reveals isoform‐specific ligand recognition and receptor recruitment in the superfamily), the interface between the cytokine TGF‐β and the receptors TGFβRI and TGFβII was identified. The selected interface between the individual receptors was divided into 9–12 amino acid peptides with one amino acid shift. On this basis, a test primary library of PI was created.

The created peptide library was used to perform docking of the receptor‐peptide complex in silico using CABS‐Dock (CABS‐dock server for flexible protein‐peptide docking, https://www.wwpdb.org) and HADDOCK (High Ambiguity Driven protein‐protein DOCKing, https://wenmr.science.uu.nl/haddock2.4) software. The docking results were compared, manually verified and used to select the first peptide library for biological activity studies. The TGF‐β1‐receptor I and II interfaces were divided into individual independent regions, from which basic structural elements were selected for further studies. At the stage of the presented pilot study, the following peptide protein inhibitors were created for the first ALDAAYCFRNVQD region of the TGF‐β1 ‐ receptor I and II interface:

1.1 ALDAAYCFR (PI1_1, Certificate of Analysis, Product Name 1_1, GenScript Biotech Corporation, Piscataway, NJ 08854, USA, see Supporting Information S1)
1.2 LDAAYCFRN (PI1_2, Certificate of Analysis, Product Name 1_2, GenScript Biotech Corporation, Piscataway, NJ 08854, USA, see Supporting Information S2)
1.3 DAAYCFRNV (PI1_3, Certificate of Analysis, Product Name 1_3, GenScript Biotech Corporation, Piscataway, NJ 08854, USA, see Supporting Information S3)
1.4 AAYCFRNVQ (PI1_4, Certificate of Analysis, Product Name 1_4, GenScript Biotech Corporation, Piscataway, NJ 08854, USA, see Supporting Information S4)
1.5 AYCFRNVQD (PI1_5, Certificate of Analysis, Product Name 1_5, GenScript Biotech Corporation, Piscataway, NJ 08854, USA, see Supporting Information S5)

The designed peptide primary inhibitors were synthesized at GenScript Biotech Corporation, 860 Centennial Ave, Piscataway, NJ 08854 USA and then tested for biological activity on MFB‐F11 cells.

2. Commercial small‐molecule non‐peptide inhibitors SD‐208 and SB‐525334

Commercial inhibitors SD‐208 (2‐[5‐Chloro‐2‐fluorophenyl)pteridin‐4‐yl]pyridin‐4‐yl‐amine), catalog no. 627536‐09‐8, manufactured by MERCK, and SB‐525334 (6‐[2‐tert‐Butyl‐5‐(6‐methyl‐pyridin‐2‐yl)‐1H‐imidazol‐4‐yl]‐quinoxaline), catalog no. 356559‐20‐1, manufactured by MERCK (Rahway, NJ 07065, USA), were used for comparative analyses of biological activity. These served as a reference marker (standard) for the efficiency of inhibition of SEAP expression (Sigma‐Aldrich) from cells of the MFB‐F11 line.

3. Cultures of MFB‐F11 and FaO cells

MFB‐F11 cells, obtained from the Stanford University School of Medicine, Stanford, CA 94305, USA were cultured in vitro. Embryonic fibroblasts from TGF‐β1−/− mice were stably transfected with a reporter plasmid consisting of TGF‐β‐responsive Smad‐binding elements coupled to a secreted alkaline phosphatase (ALP) reporter gene (SBE‐SEAP, Sigma‐Aldrich).

MFB‐F11 cells were cultured according to the protocol of the Tony Wyss‐Coray lab, updated 3/9/11 from the Stanford University School of Medicine, Stanford, CA 94305, USA. The following materials were used in the culture: DMEM (Cat# 21041‐025, GIBCO), PBS (Cat# D8537, Sigma‐Aldrich), for washing cells before treatment), FBS (Cat#F7524, fetal bovine serum, Sigma‐Aldrich), Hygromycin (Cat#10687010, INVITROGEN, Waltham, MA 02451, USA), Pen‐Strep (Cat#11074440001, penicillin/streptomycin, INVITROGEN) and TGF‐β [Recombinant Human TGF‐β1 (HEK293 derived)]. All the above materials are original and they were purchased from recommended manufacturers.

D10: DMEM supplemented with 10% FBS and 1x Pen‐Strep.

Protocol steps for performing the MFB‐F11 culture procedure: Cells

- Thaw a vial of frozen cells quickly in a 37^o^C water bath and transfer into a 100 mm culture dish containing 9 mL D10. Place the dish into a 37^o^C tissue culture incubator supplied with 5% CO_2_.
- The next day, replace the medium with fresh D10, and add 125 μg/mL hygromycin.
- Passage cells when they are confluent. Cells could be seeded at 1:10 or even higher dilutions. Cells need to be passaged twice a week if seeded at 1:10 dilution.
- For experiments, seed cells at 20,000–40, 000 cells/well in 96‐well plate in D10 or scale up for other culture vessels. The next day, wash the cells twice with DPBS, change the medium to DMEM/Pen‐Strep and add treatment.

The Stanford University School of Medicine, Stanford, CA 94305, USA Note Under the abovementioned culture conditions, this cell line has been tested for 80 passages without any obvious loss of response to recombinant TGF‐β1 as determined by the SEAP assay.

Commercial FaO cells, which were purchased from Sigma (Cat#85061112, Sigma‐Aldrich), were used for the culture procedure. We used the following cell culture solutions: Medium F12‐K (Cat# 21127‐022, GIBCO), supplemented with 10% FBS (Cat#F7524, fetal bovine serum, Sigma‐Aldrich), pen‐strep (Cat#11074440001, penicillin/streptomycin, INVITROGEN) and gentamicin (Cat# 15710‐064, GIBCO). Other cell culture protocol steps are similar to those contained in the cell culture protocol provided by the manufacturer.

4. Assessment of PI toxicity by flow cytometry‐based apoptosis assay

The data were recorded on a Canto II flow cytometer (Becton Dickinson). The data were visualized and quantified by constructing a dot‐plot using the BD FACSDiva software. The cytometric analysis was performed by a professional analyst holding a PhD degree under the supervision of the head of the Research Laboratory—CoreLab, Lodz 92–215, Lodzkie Voivodeship, Poland.

Cytometric analysis of the response to TGF‐β1 by determining necrosis/apoptosis of MFB‐F11 and FaO cells was performed. The following were determined: Q1—necrotic cells; Q2—late apoptotic cells; Q3—healthy cells; and Q4—early apoptotic cells.

5. Biological evaluation of TGF‐β‐Smad pathway activation following the use of PI with the quantitative real‐time polymerase chain reaction (qRT‐PCR) method.

RNA was isolated from cells harvested from MFB‐F11 cell cultures. Genetic material was isolated using the Total RNA Mini kit (Cat# 031–100, A&A Biotechnology, Gdansk 80–299) according to the guidelines provided by the manufacturer. The extracted RNA was analyzed with agarose gel electrophoresis and only cases with preserved 28 S, 18 S and 5 S ribosomal RNA bands, indicating good RNA quality, were used in the study. The amount of purified RNA was determined using spectrophotometry at 260 nm in a Nanodrop analyzer (ND‐100; Nanodrop Technologies). The purity was verified according to the ratio of 260/280 nm measurements, and values between 1.8 and 2.1 indicated that the quality of the obtained RNA was optimal and suitable for a qRT‐PCR. cDNA synthesis reactions were performed using the ImProm‐II™ Reverse Transcription System kit (Cat# A3800, Promega). Expression analysis of the studied genes was performed in the Department of Medical Biochemistry of the Medical University of Lodz, Lodzkie Voivodeship, Poland.^10^, ^22^, ^23^, ^24^

Expression was analyzed for the following genes:

‐ Smad family genes, encoding proteins serving as signal transducers and transcription modulators, which mediate many signaling pathways:
  ∗ Smad2 (Smad family member 2),
  ∗ Smad3 (Smad family member 3),
  ∗ Smad4 (Smad family member 4).
‐ JNKs, c‐Jun N‐terminal kinases (JNKs), and kinases that bind and phosphorylate c‐Jun on Ser‐63 and Ser‐73 within its transcriptional activation domain:
  ∗ JNK1/2 (JNKs kinase signaling pathways are involved in control of immune responses).

For the purpose of internal control, the 18 S rRNA (#4333760F, Life Technologies) gene was used, which demonstrates that expression in the tested samples is a constant. Appropriate TaqMan probes that do not react with genomic DNA were chosen for the eight genes and the control gene (18 S rRNA) was selected for the analysis. They are presented in Table 1.

**TABLE 1:** Analyzed genes and the applied TaqMan probes.

Studied gen	UniGene ID	Assay ID	Primer annealing temperature
SEAP	2,828,033	F_TCATCCCAGTTGAGGAGGAG	59°C
		R_GATCCTGGCAGCTGTCACC	
18 S rRNA	100,008,588	4333760F	The same as for the studied gene
Smad2	Hs.12253	Hs00998187_m1	58°C
Smad3	Hs.727986	Hs00969210_m1	60°C
Smad4	Hs.75862	Hs00929647_m1	60°C
JNK1	Hs.446850	Hs00255559_m1	60°C
p44	Hs.204773	Hs00560452_g1	56°C

For each sample, CT (threshold cycle) values were calculated with the use of Mx‐Pro software. The qRT‐PCR amplification of each gene was compared to that of 18 S rRNA, a house‐keeping reference gene and ΔCT values were determined (ΔCT = CT, GENE ‐ CT, 18 S rRNA). The qRT‐PCR data were automatically calculated using the data analysis module. The results were analyzed according to the 2‐ΔΔCT method with the assumption of 100% reaction yield. Validation of PCR efficiency was performed using a standard curve. The complementary DNA (cDNA) was subjected to real‐time quantitative PCR using gene‐specific primers for the studied genes and 18 S rRNA with the use of the TaqMan Sonds® & Master Mixes for qRT‐PCR (Biotium, Inc.).^10^, ^22^, ^23^, ^24^, ^25^, ^26^, ^27^

The analysis of expression of selected genes was performed using a Real‐Time PCR Optical Thermocycler manufactured by Biometra (Biometra Biomedizinische Analytik GmbH) using commercial TaqMan probes. TaqMan probes are hybridization probes designed to increase the specificity of Real‐Time PCR reactions.

Assays were performed in two repetitions for each sample. Averaged Ct values for the two replicates make up the results of the study. Ct is identified as the number of amplification cycles of the PCR product in which the fluorescence level of the dye exceeds the threshold called the limit cycle. Considering the Ct value, it is possible to analyze the amount of baseline cDNA for selected genes in the studied sample, and thereby to analyze their expression.^10^, ^22^, ^23^, ^24^, ^25^, ^26^, ^27^

### 3.1 Data analysis

The statistical analyses were conducted by a professional statistician using licensed computing software. A statistical test called one‐way ANOVA analysis with Dunnett's two‐sided post‐hoc test was applied. An analysis of mRNA expression changes of the studied proteins was performed by assessing ddCt and compared to the newly designed peptide inhibitors (IPs) with the existing commercial small‐molecule inhibitors SB‐525334 and SD‐208, as detailed in the tables in the Results section. *p*‐values below 0.05 were considered statistically significant. The analysis was performed using the STATISTICA 13.1 software (StatSoft) and R Software version 3.6.1 (R Core Team 2019).

### 3.2 Ethical approval

The study was approved by the local Ethics Committee (consent of the Research Review Board of the Medical University of Lodz, Lodz, Poland; no. RNN/31/14/KE).

## 4 RESULTS

The TGF‐β1‐receptor I and II interface was subdivided into individual independent regions, from which primary structural elements were selected for further study (pilot study):

The peptide library created for Region 1: Region 1 ALDAAYCFRNVQD

1.1 ALDAAYCFR (IP1_1)
1.2 LDAAYCFRN (IP1_2)
1.3 DAAYCFRNV (IP1_3)
1.4 AAYCFRNVQ (IP1_4)
1.5 AYCFRNVQD (IP1_5)

**FIGURE 1:**
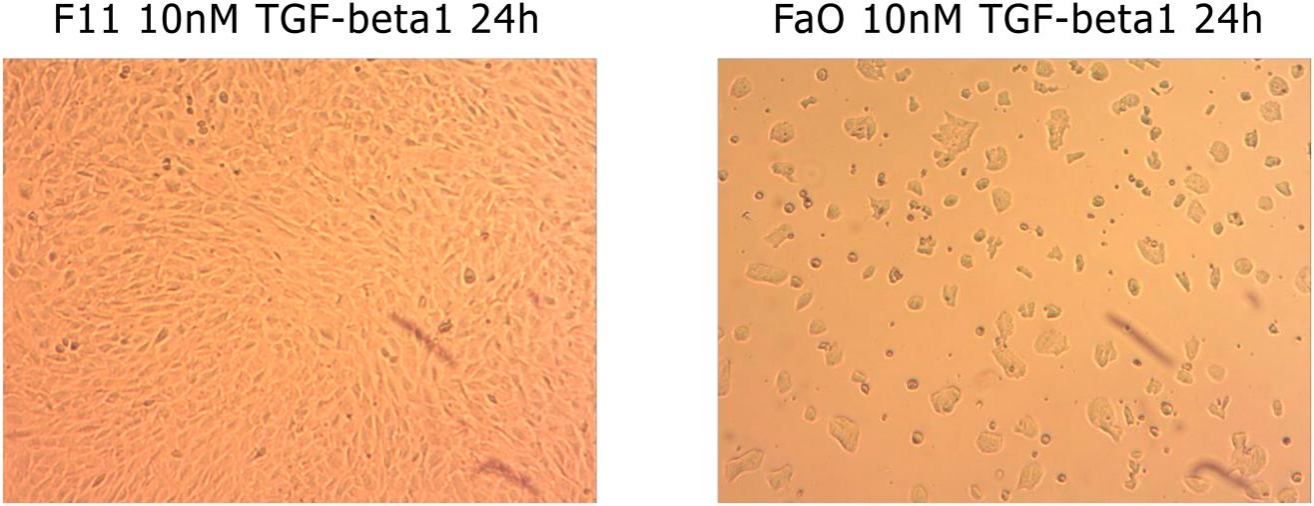
24 h incubation of cells of MFB‐F11 and FaO lines with TGF‐β1 at a concentration of 10 nM. The difference in response to TGF‐β1 is evident here, and it manifests as a disparity in the cell growth of the respective lines (MFB‐F11 without a response).

**FIGURE 2:**
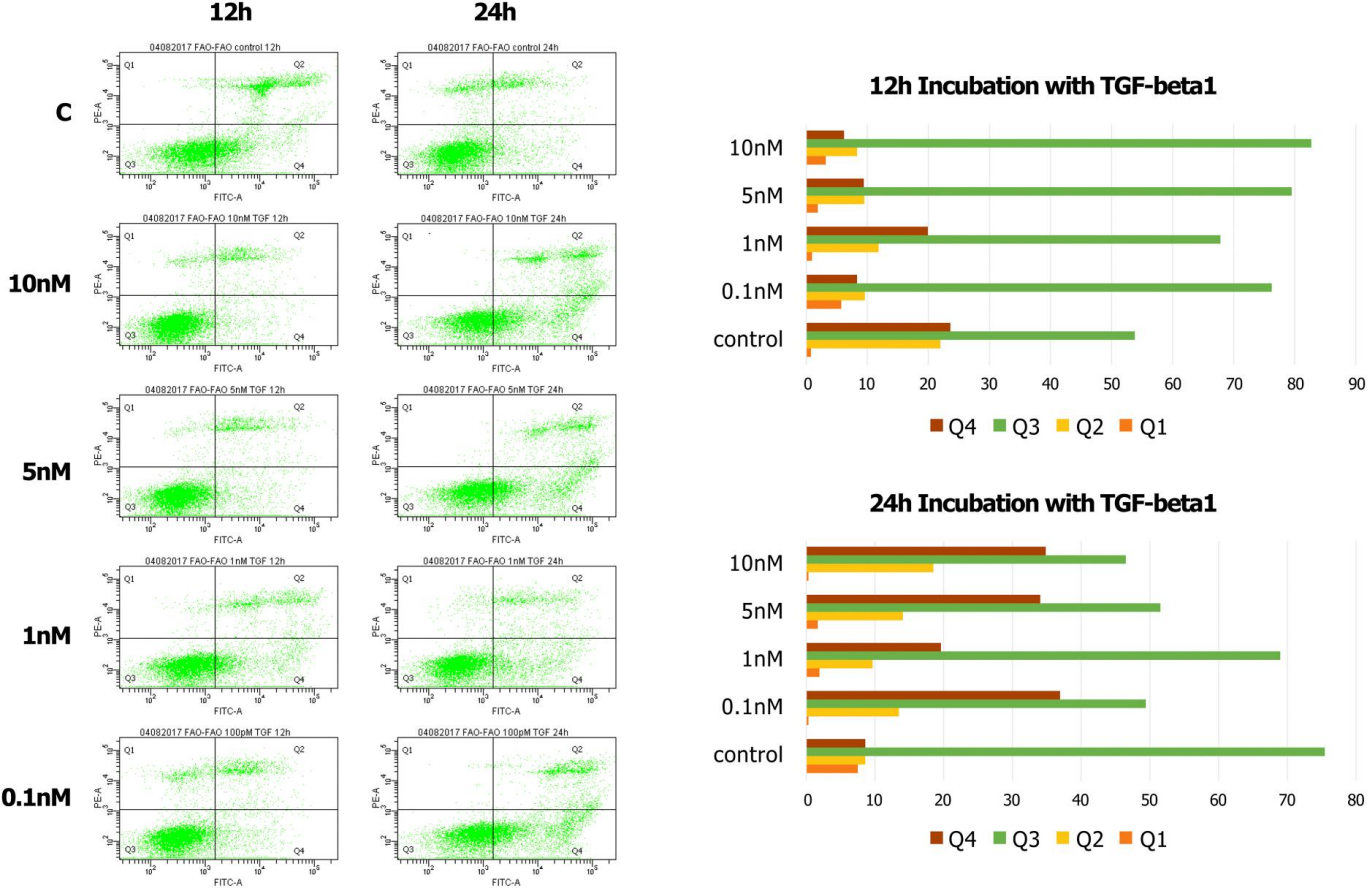
The cytometric analysis of the response to TGF‐β1 by determining necrosis/apoptosis of FaO line cells: Q1—necrotic cells; Q2—late apoptotic cells; Q3—healthy cells; and Q4—early apoptotic cells. For the FaO TGF‐β1 line, after 24 h incubation, there is a clear increase in the population of apoptotic cells in comparison with the control.

**FIGURE 3:**
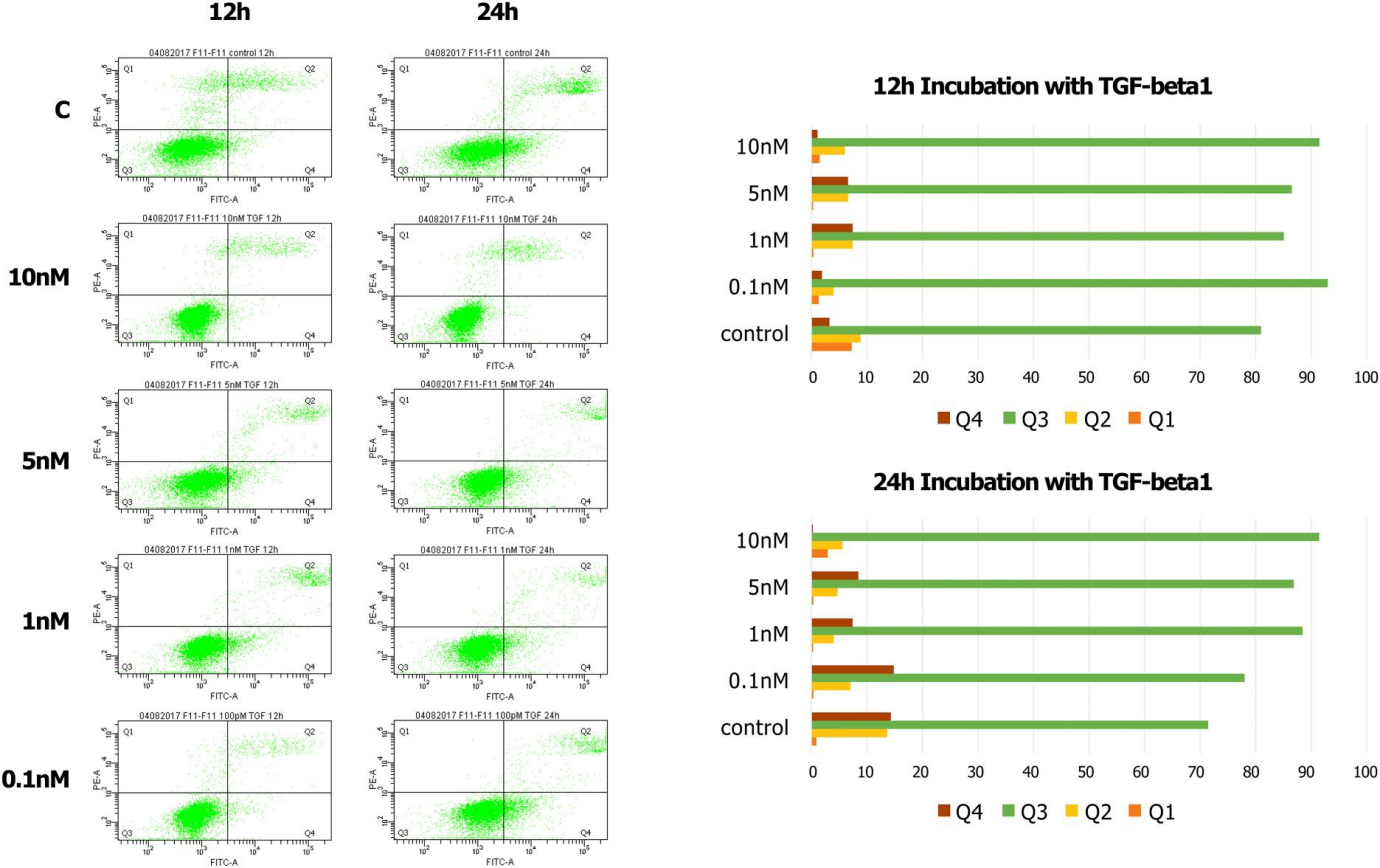
The cytometric analysis of the response to TGF‐β1 by determining necrosis/apoptosis of the MFB‐F11 cell line: Q1—necrotic cells; Q2—late apoptotic cells; Q3—healthy cells; and Q4—early apoptotic cells.

A series of experiments assessing necrosis/apoptosis using flow cytometry with annexin V staining (apoptotic cells) and propidium iodide (dead cells) was performed, confirming that cells of the FaO line get damaged during experimental cell cultures within 24 h in increasing concentrations of the cytokine TGF‐β, as shown in Figures 1, 2, 3 (Q1—necrotic cells, Q2—late apoptotic cells, Q3—healthy cells, Q4—early apoptotic cells). We thereby confirmed the usefulness and efficacy of the FaO cell line in assessing the toxicity of newly designed peptide inhibitors (Figures 1, 2, 3).

For the MFB‐F11 line, TGF‐β1 stimulates cell growth and has an anti‐apoptotic effect.

**FIGURE 4:**
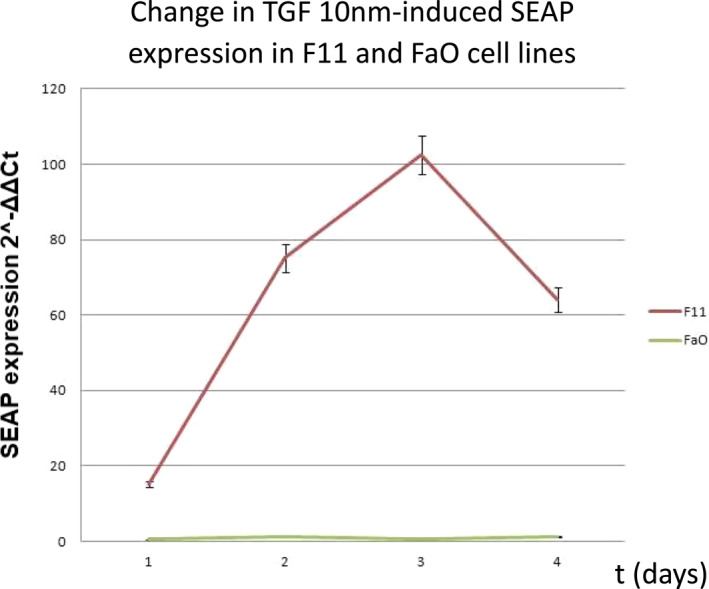
Changes in the expression of the SEAP reporter gene in the MFB‐F11 line after 24, 48, 72 and 96 h of stimulation with 10 nM TGF‐β1. The increase in the expression is noticeable as early as after 24 h and it reaches its maximum value after 72 h (the red line) compared to the negative control (cells of the FaO line, the green line).

**FIGURE 5:**
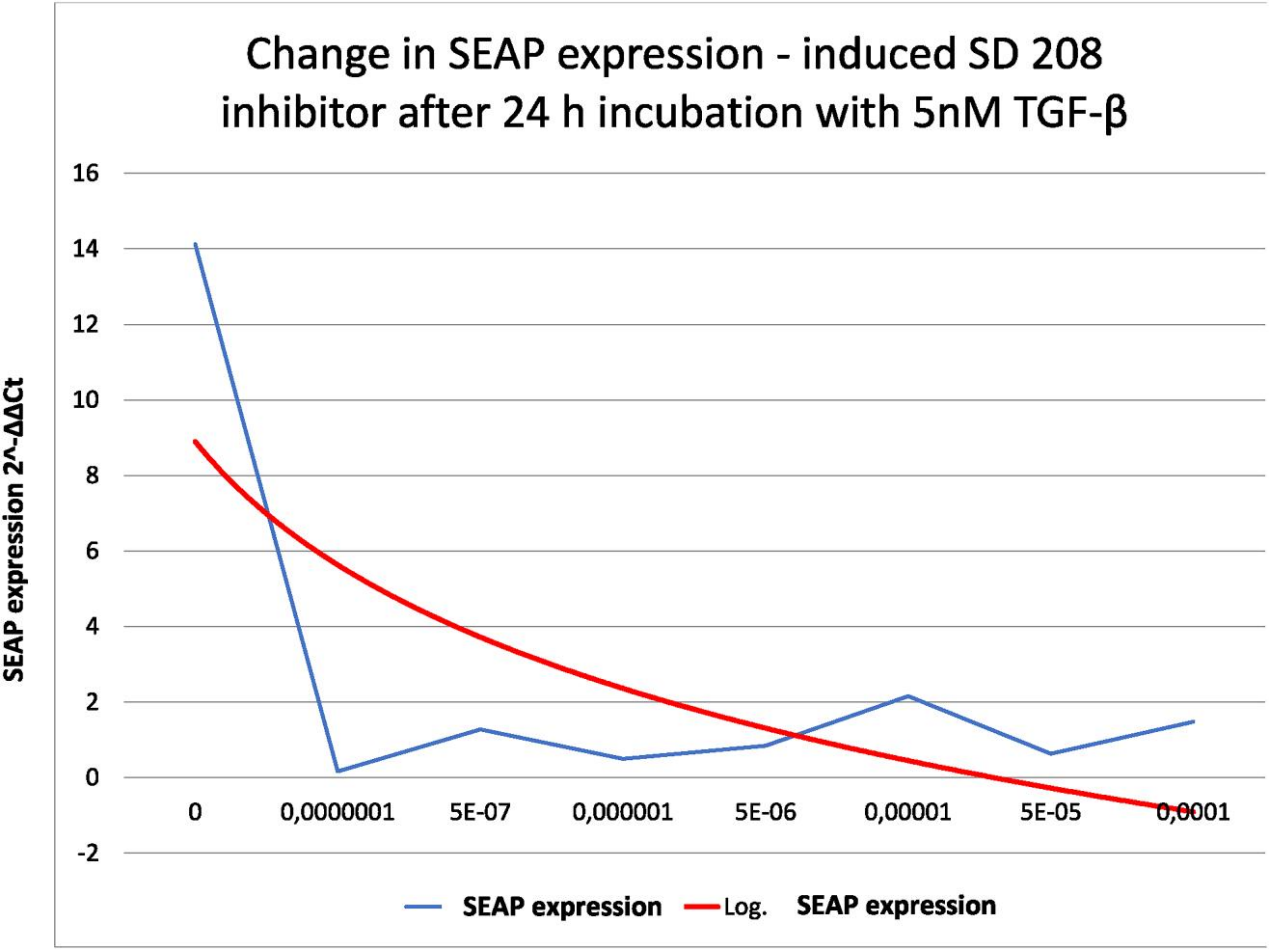
Inhibition of SEAP expression from cells of the MFB‐F11 line under the influence of the commercial inhibitor SD‐208 (2‐(5‐Chloro‐2‐fluorophenyl) pteridin‐4‐yl]pyridin‐4‐yl‐amine).

**FIGURE 6:**
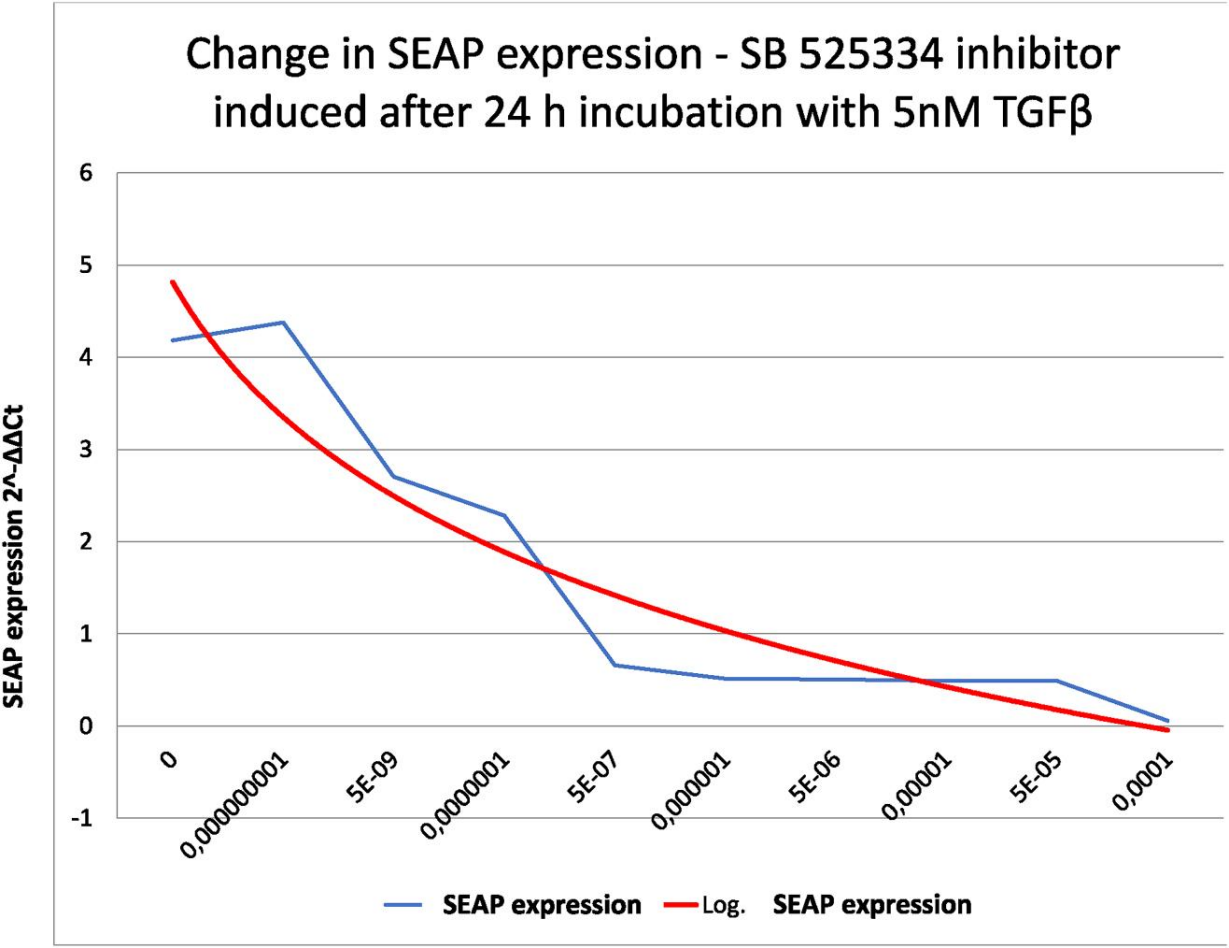
Inhibition of SEAP expression from cells of the MFB‐F11 line induced by the commercial inhibitor SB‐525334 (6‐[2‐tert‐Butyl‐5‐(6‐methyl‐pyridin‐2‐yl)‐1H‐imidazol‐4‐yl]‐quinoxaline).

Besides, pre‐cultures of MFB‐F11 (showing SEAP reporter gene expression after exposure to TGF‐β—Figure 4) and FaO (undergoing apoptosis after exposure to TGF‐β—Figure 4) cell lines were incubated. We verified the effect of two commercial small‐molecule inhibitors (SD‐208 and SB‐525334) of type I TGF‐β receptor, which will serve as positive controls in our subsequent measurements (Figures 5 and 6). The analysis was performed by flow cytometry (for the FaO line) and qRT‐PCR (for the MFB‐F11 line).

**FIGURE 7:**
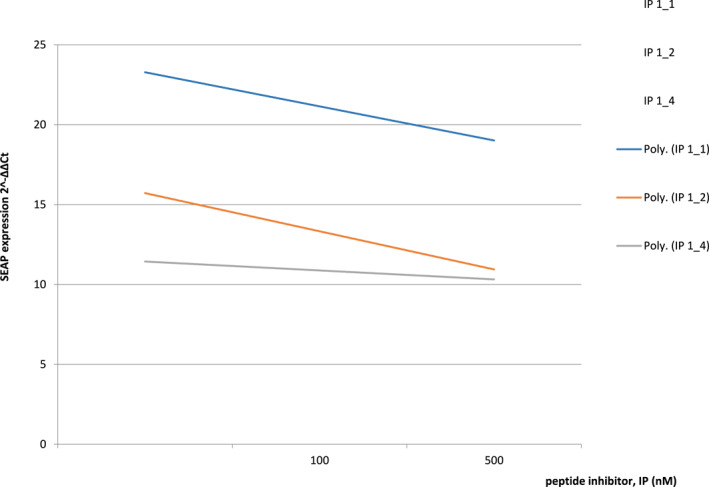
Inhibition of SEAP expression from MFB‐F11 line cells induced by peptide inhibitors investigated peptides (IP) 1_1, IP 1_2 and IP 1_4 after 24 h stimulation with 5 nM TGF‐β1.

For the following selected inhibitors, IP1_1,1_2 and 1_4, the ability of SEAP reporter gene expression was demonstrated, thereby confirming their effectiveness in blocking the TGF‐β receptor, as shown in Figure 7 (Other inhibitors are being studied; we present only selected results obtained from the pilot study).

**FIGURE 8:**
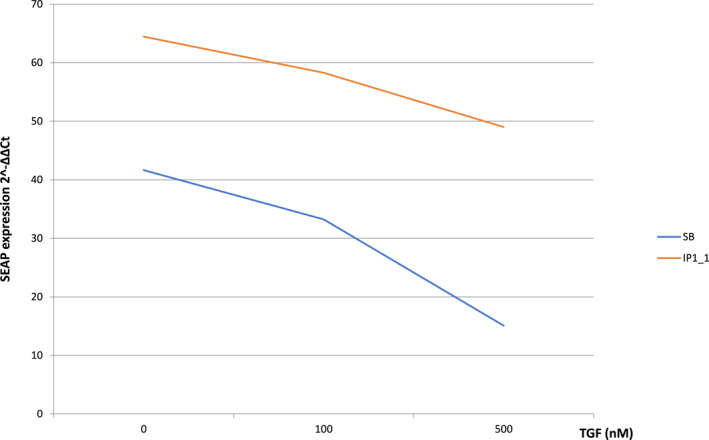
Comparative analysis of SEAP expression from MFB‐F11 line cells induced by small‐molecule inhibitor SB‐525334 (6‐[2‐tert‐Butyl‐5‐(6‐methyl‐pyridin‐2‐yl)‐1H‐imidazol‐4‐yl]‐quinoxaline) and peptide investigated peptides (IP) 1_1 after 24 h stimulation with 5 nM TGF‐β1.

Following the objectives of the research project, we compared the activities of the pre‐tested and only selected peptide inhibitors with commercial small‐molecule inhibitors. Figure 8 shows changes in the SEAP reporter gene expression between the newly designed inhibitor protein IP1_1 and commercial inhibitor SB‐525334 (Figure 8).

We analyzed mRNA expression changes of the studied proteins by assessing ddCt and comparing the activity of newly designed peptide inhibitors (IPs) with the existing commercial small‐molecule inhibitors SB‐525334 and SD‐208, as it is shown in Tables 2, 3, 4, 5 (other inhibitors are being studied; we present only selected results obtained from the pilot study). Of the 5 new IPs used in the study, only three peptides that met the criteria for biological functionality were selected. The investigated peptides (IP) peptides selected through molecular activity studies were compared with the existing commercial inhibitors SB‐525334 and SD‐208. The newly designed IP peptides (IP1_2, 1_3, 1_5) appeared to demonstrate comparable or even higher effectiveness in inhibiting pro‐fibrotic cytokines and their TGFβRI/TGFβRII—TGF‐β—Smad signaling pathway proteins (see Tables 2, 3, 4, 5). This important finding allows us to conclude that the newly designed IPs demonstrate higher efficacy in blocking the TGFβRI/TGFβRII receptor complex than previous small‐molecule compounds (SB‐525334 and SD‐208).

**TABLE 2:** Smad2.

Studied IP [50 μM]	I reference [50 nM]	ddCt	*p*‐value
IP1_2	SB‐525334	−12.2	0.0000
IP1_3	SB‐525334	−10.5	0.0001
IP1_5	SB‐525334	−2.4	0.3410
IP1_2	SD‐208	−8.0	0.0008
IP1_3	SD‐208	−6.3	0.0040
IP1_5	SD‐208	1.8	0.6041

*Note*: IP, newly designed peptide inhibitor; I, small‐molecule inhibitor, commercial (reference).

**TABLE 3:** Smad3.

Studied IP [50 μM]	I reference [50 nM]	ddCt	*p*‐value
IP1_2	SB‐525334	−11.9	0.0001
IP1_3	SB‐525334	−9.5	0.0004
IP1_5	SB‐525334	0.2	1.0000
IP1_2	SD‐208	−12.0	0.0001
IP1_3	SD‐208	−9.5	0.0004
IP1_5	SD‐208	0.2	1.0000

*Note*: IP, newly designed peptide inhibitor; I, small‐molecule inhibitor, commercial (reference).

**TABLE 4:** Smad4.

Studied IP [50 μM]	I reference [50 nM]	ddCt	*p*‐value
IP1_2	SB‐525334	−11.3	0.0002
IP1_3	SB‐525334	−9.3	0.0006
IP1_5	SB‐525334	0.9	0.9677
IP1_2	SD‐208	−11.5	0.0002
IP1_3	SD‐208	−9.4	0.0006
IP1_5	SD‐208	0.8	0.9860

*Note*: IP, newly designed peptide inhibitor; I, small‐molecule inhibitor, commercial (reference).

**TABLE 5:** Jun N‐terminal kinase.

Studied IP [50 μM]	I reference [50 nM]	ddCt	*p*‐value
IP1_2	SB‐525334	−15.3	0.0001
IP1_3	SB‐525334	−13.4	0.0002
IP1_5	SB‐525334	−0.1	1.0000
IP1_2	SD‐208	−16.5	0.0001
IP1_3	SD‐208	−14.6	0.0001
IP1_5	SD‐208	−1.3	0.9684

*Note*: IP, peptide inhibitor, newly designed; I, small‐molecule inhibitor, commercial (reference).

## 5 DISCUSSION

Studies on the use of different types of inhibitors of signaling pathways for TGF‐β have been conducted. These inhibitors comprise small‐molecule and peptide inhibitors, including monoclonal antibodies.^28^ Studies using peptide P144 (TSLDASIIWAMMQN), which blocks the extracellular domain of TGFβRIII, thereby preventing its interaction with the cytokine, have demonstrated that the inhibitor may potentially serve as an anti‐fibrotic agent in one model of chronic liver injury in rats.^29^ P17 (KRIWFIPRSSWYERA) appeared to be another PI for TGF‐β. It has been shown to block the effects of the TGF‐β1 cytokine inducing the expression of genes responsible for collagen production.^30^ In vitro, P17 inhibited TGF‐β‐stimulated expression of fibrosis‐promoting substances in tissues, whereas in vivo, this peptide reduced lung fibrosis in bleomycin‐treated mice.^31^

In our study, we focused on the mechanism of action of the TGF‐β cytokine family, which first binds to the TGFβRII receptor (dimer), resulting in the recruitment of the TGFβRI receptor (dimer). The complex thus formed leads to the phosphorylation of the kinase domain of TGFβRI, which in turn results in the activation of the Smad pathway.^9^, ^10^, ^11^ This is therefore a targeted pathway for research into the application of peptide inhibitors in blocking the TGF‐β‐Smad signaling pathway, as shown in our pilot study.

Analyses of the functional role of selected TGF‐β cytokine homologous molecules conducted so far as part of the pilot study have demonstrated that newly designed peptide inhibitors for the region I inhibit gene expression of Smad‐dependent and Smad‐independent pathways, which indicates that they are potent to block the TGFβRI/TGFβRII receptor complex in vitro (Figures 7 and 8, Tables 2, 3, 4, 5).

Results of experiments carried out on the design and synthesis of TGFβRI/TGFβRII inhibitors allowed us to identify and describe key regions of the TGF‐β ‐ TGFβRI/TGFβRII interface, that is, for Region 1 ALDAAYCFRNVQD. Analytical work for the description of Region 2 of CAGACPYLWSSDTQHSR is currently being conducted.

The following five peptide inhibitors were investigated for Region 1: 1_1 ALDAAYCFR, 1_2 LDAAYCFRN, 1_3 DAAYCFRNV, 1_4 AAYCFRNVQ, 1_5 AYCFRNVQD. We are presenting partial results. With regard to IP1_2,1_3 and 1_5 Region 1 peptides tested for TGFβRI/TGFβRII, the cytokine‐receptor relay signal was reduced in comparison to controls (SB‐525334 and SD‐208 inhibitors) after adding the newly designed inhibitors. It was confirmed that these IP inhibitors reduce mRNA expression of the Smad2, Smad3, Smad4 and JNK1 genes (Figure 7 and Tables 2, 3, 4, 5). Other peptide inhibitors are currently being studied, and we present only selected results of the pilot study.

The efficacy of the mentioned inhibitors in blocking TGF‐β was also observed in the FaO line. The addition of TGF‐β to the FaO line leads to morphological changes and induction of apoptosis.^32^ Application of newly designed peptide inhibitors after prior addition of TGF‐β to FaO line cultures did not change the life‐span of the FaO line. Besides, no toxic effect was observed after self‐administration of the peptides (IP) to FaO line cultures (Figures 1, 2, 3).

Results of the pilot study indicate there is a need to conduct further research in the presented laboratory techniques, modify the structure of the existing peptides and analyze inhibited proteins in the Smad and non‐Smad dependent signaling pathways in the cellular response to TGF‐β cytokine impact on them with the use of other research methods.

It should be pointed out that all experiments were double‐checked against a positive control and a negative control. Moreover, assays were performed each time for commercial inhibitors SB‐525334 and SD‐208, which were treated, as reference (standard curves) for the purpose of evaluation of new peptide inhibitors (Tables 2, 3, 4, 5). Unfortunately, only selected results of the experiments performed so far are presented. The authors are conducting further molecular studies and the remaining results are still being prepared to obtain more data from this pilot study.

IP peptides 1_2, 1_3 and 1_5, selected through biological activity studies, should then be subjected to much more intensive peptide‐receptor docking again in the future (using the abovementioned methods in combination with adjustment MD) in order to obtain the most precise possible picture of docked molecules. This would enable us to estimate the chances of improving the activity by truncation, elongation, or mutagenesis of the IP.

The newly designed IP peptides should be subjected to further in silico verification studies using CABS‐Dock and HADDOCK computer software. In the future, they might be used while preparing further libraries for studies on biological activity. Further studies should focus on improving the activity of inhibitors by synthesizing new peptides with slightly different amino acid structures.

Research into the synthesis of targeted, specific and non‐toxic peptide inhibitors for TGFβRI/TGFβRII, as it was presented in the above pilot study (Figures 7 and 8 and Tables 2, 3, 4, 5), can help invent new therapeutic substances to prevent the development of chronic inflammation in many organs as well as its complications, particularly organ remodeling, which in turn leads to remodeling of many tissues. Adverse effects of the environment on various organs, both through specific (haptens, antigens, allergens) and non‐specific (biological and non‐biological) factors, which have recently become a growing problem in modern civilization due to increasing air pollution, could thus be prevented.

## 6 LIMITATIONS

The results should be verified by other physico‐chemical and biochemical methods. The activity of secreted SEAP, added to MFB‐F11 IP and TGF‐β1, could be further analyzed by spectrophotometry.

Calorimetric assessment of the absorbance of SBE‐SEAP (secreted ALP) with the use of SIGMAFAST™ p‐Nitrophenyl phosphate could also be made to verify preliminary data obtained from the pilot study.

Stimulation of MFB‐F11 cells contributed to an increase in the amount of secreted ALP in the culture medium. This enzyme catalyzes the hydrolysis of p‐nitrophenyl phosphate at pH = 10.4, releasing p‐nitrophenyl and phosphate. The rate of p‐nitrophenol formation measured using the photometric method is proportional to the concentration of phosphatase in the sample. Quantitative determination of ALP was performed according to the SIGMAFAST™ p‐Nitrophenyl phosphate N2770‐50SET kit (Sigma‐Aldrich, Cleveland, OH 44125, USA).

The dissociation constant (Kd) of the PI‐TGFβRI/TGFβRII receptor should also be assessed by intermolecular interaction analysis using microscale thermophoresis (MST) and surface plasmon resonance (SPR) techniques to further analyze interactions of selected peptides. Subsequent phases of the study should consist of repeating loop phases [analysis of selected peptide interactions‐intensive peptide‐receptor docking (using the above methods in combination with MD adjustment) several times until a PI with maximum binding constant to the TGFβRI/TGFβRII receptor has been identified.

Studies using techniques other than those applied in the pilot study should also include a more extensive assessment of changes in the expression and levels of Smad‐dependent and Smad‐independent pathway proteins, including but not limited to: Smad—MAP kinase—p38, JNK and Ersk1/2 under the influence of the newly designed IPs.

## 7 CONCLUSIONS

An analysis of peptide TGF‐β inhibitors (designed and synthesized in the pilot study) blocking the TGFβRI/TGFβRII—Smad—JNK protein system enabled us to show the way we should follow while conducting further research in this area. This research should involve the determination of the strength of the peptide—and TBRII complexes (human type I TGF‐β receptor and human type II TGF‐β receptor, recombinant proteins) with the use of the SPR technique, conducting bioinformatics analyses estimating the chance of improving the IP activity by either truncation, elongation or mutagenesis of the tested peptides as well as conducting studies evaluating the inhibition of the TGFβRI/TGFβRII—Smad—MAP kinase—p38, JNK and Ersk1/2 protein system, which can effectively reduce the mRNA expression for many pro‐fibrotic proteins.

## AUTHOR CONTRIBUTIONS

**Marynowski Mateusz**: Conceptualization (supporting); investigation (equal); project administration (equal); resources (equal); software (equal). **Karbownik Michal Seweryn**: Conceptualization (equal); data curation (equal); formal analysis (equal); methodology (equal); software (equal); supervision (equal); validation (equal). **Szemraj Janusz**: Conceptualization (equal); project administration (equal); supervision (equal). **Kuna Piotr**: Funding acquisition (equal); supervision (equal). **Michał Gabriel Panek**: Conceptualization (equal); data curation (equal); formal analysis (equal); funding acquisition (equal); investigation (equal); methodology (equal); project administration (equal); resources (equal); software (equal); supervision (equal); validation (equal); visualization (equal); writing—original draft (equal); writing—review & editing (equal).

## CONFLICT OF INTEREST STATEMENT

The authors declare that there are no conflicts of interest regarding the publication of this article.

## Supporting information

Supporting Information S1

Supporting Information S2

Supporting Information S3

Supporting Information S4

Supporting Information S5

## Data Availability

The original contributions presented in the study are included in the article/Supplementary Material. Further inquiries can be sent to the corresponding author.
