# Supplementary material for: Assessment of the effectiveness of the peptide inhibitor homologous to the transforming growth factor β cytokine blocking the TGFβRI/TGFβRII receptor complex—pilot study
Source: Clin Transl Allergy. 2023 Dec 31;14(1):e12320. doi: 10.1002/clt2.12320 (PMC10758017; doi:10.1002/clt2.12320)
Supplement: Supplementary file 1 — Supporting Information S1 [file CLT2-14-e12320-s005.pdf]

CERTIFICATE OF ANALYSIS

Product Name	1_1
Order ID	U8824CK230_1
Lot No.	93622750001/PE9097
Sequence	ALDAAYCFR
Modification	N/A
Length	9AA
Storage	-20°C
Recommended Solvent*	Ultrapure water
comments	TFA salt

Test Items	Specifications	Results
Molecular Weight	Theoretical MW: 1029.18	Consistent
HPLC purity	≥75.0%	97.9%
Appearance	White lyophilized powder	Conforms
Gross Weight	1-4 mg	4.3mg

*Note: Above recommended solvents for reference only. If there is any request for detailed dissolution conditions,we suggest you choose our 'Peptide Solubility Test Service'.

Caution:
For laboratory or further manufacturing use only. Not intended for household use. If you have any questions about the Certificate of Analysis, please contact our customer service representative at 1-877-436-7274 (Toll-Free), or 1-732-885-9188.

Certified by: Gary Chen Date:12-08-2017
Peptide Production Director

Thank you for your patronage to our Peptide services! To maintain this working relationship, we shall be grateful if you can add our webpage URL into your lab website. As a token of appreciation, you will be rewarded by 1,000 EZcoupon™ points. For more information, please contact us by e-mail at web@genscript.com
