# Supplementary material for: Assessment of the effectiveness of the peptide inhibitor homologous to the transforming growth factor β cytokine blocking the TGFβRI/TGFβRII receptor complex—pilot study
Source: Clin Transl Allergy. 2023 Dec 31;14(1):e12320. doi: 10.1002/clt2.12320 (PMC10758017; doi:10.1002/clt2.12320)
Supplement: Supplementary file 3 — Supporting Information S3 [file CLT2-14-e12320-s004.pdf]

# CERTIFICATE OF ANALYSIS

| | |
|---|---|
| **Product Name** | 1_3 |
| **Order ID** | U8824CK230_3 |
| **Lot No.** | 93622750003/PE9099 |
| **Sequence** | DAAYCFRNV |
| **Modification** | N/A |
| **Length** | 9AA |
| **Storage** | -20°C |
| **Recommended Solvent*** | Ultrapure water |
| **comments** | TFA salt |

| Test Items | Specifications | Results |
|---|---|---|
| **Molecular Weight** | Theoretical MW: 1058.17 | Consistent |
| **HPLC purity** | ≥75.0% | 95.3% |
| **Appearance** | White lyophilized powder | Conforms |
| **Gross Weight** | 1-4 mg | 4.7mg |

*Note: Above recommended solvents for reference only. If there is any request for detailed dissolution conditions,we suggest you choose our 'Peptide Solubility Test Service'.

**Caution:**
For laboratory or further manufacturing use only. Not intended for household use. If you have any questions about the Certificate of Analysis, please contact our customer service representative at 1-877-436-7274 (Toll-Free), or 1-732-885-9188.

Certified by: Garg Chen
Peptide Production Director

Date:12-05-2017

Thank you for your patronage to our Peptide services! To maintain this working relationship, we shall be grateful if you can add our webpage URL into your lab website. As a token of appreciation, you will be rewarded by 1,000 EZcoupon™ points. For more information, please contact us by e-mail at web@genscript.com
